# Immune and non-immune hydrops fetalis in a Saudi tertiary center: etiologies, antenatal predictors, perinatal outcomes, and one-year survival in a seven-year cohort

**DOI:** 10.3389/fped.2026.1693325

**Published:** 2026-05-04

**Authors:** Kamal Ali, Abdullah Alharbi, Saleh Alshaibi, Abdulrahman Almunif, Sadeem Alnami, Alwateen Al Abdullah, Usama Alharbi, Nadia Al Ghilan, Saad M. Alshamrani, Mohammed Khan, Abdulaziz Homedi, Ibrahim Ali, Saif Alsaif

**Affiliations:** 1 Neonatal Intensive Care Department, Women Health Hospital, King Abdulaziz Medical City-Riyadh, Ministry of National Guard Health Affairs, Riyadh, Saudi Arabia; 2 King Abdullah International Medical Research Center, Riyadh, Saudi Arabia; 3 College of Medicine, King Saud Bin Abdulaziz University for Health Sciences, Riyadh, Saudi Arabia; 4 Department of Maternal Fetal Medicine, Women Health Hospital, King Abdulaziz Medical City-Riyadh, Ministry of National Guard Health Affairs, Riyadh, Saudi Arabia

**Keywords:** birth prevalence, immune hydrops, intrauterine transfusion, non-immune hydrops fetalis, pleural effusion

## Abstract

**Background:**

Hydrops fetalis encompasses diverse fetal disorders with high perinatal mortality, and contemporary regional data are limited.

**Methods:**

We conducted a single-center retrospective cohort study at a Saudi tertiary referral hospital (2016–2022). Pregnancies with antenatal hydrops (≥2 fluid-filled compartments) that were delivered at our center were included and classified as immune or non-immune hydrops fetalis (NIHF). For NIHF, multivariable logistic regression identified predictors of (i) live birth vs. intrauterine fetal death (IUFD)/termination in the antenatal cohort and (ii) one-year survival among live-born infants.

**Results:**

Of 63,000 deliveries, the hospital-based birth prevalence of antenatally diagnosed NIHF and immune hydrops was 1.84/1,000 and 0.22/1,000, respectively. Among live births, the prevalence was 0.65/1,000 (NIHF) and 0.16/1,000 (immune). In the NIHF antenatal cohort (*n* = 116), the outcomes were IUFD 56.9% (66/116), termination 7.8% (9/116), and live birth 35.3% (41/116). Of the 41 live-born infants with NIHF, 43.9% (18/41) survived to one year of age. In the multivariable analysis, the antenatal factors that were independently associated with IUFD/termination were earlier gestational age at diagnosis [adjusted odds ratio [aOR] 1.20 per week earlier, 95% confidence interval [CI] 1.09–1.31; *p* < 0.001] and fetal pleural effusion (aOR 6.11, 95% CI 2.07–17.98; *p* = 0.001). Among live-born infants with NIHF, gestational age at delivery was the sole independent predictor of one-year survival (aOR 1.55 per additional week, 95% CI 1.09–2.22; *p* = 0.016). In the immune hydrops subgroup (*n* = 14), most pregnancies received intrauterine transfusion, and the one-year survival was 71.4% (10/14). Among the ten live-born immune cases, postnatal management was intensive, with intravenous immunoglobulin (IVIG) administration in 100% of cases, double-volume exchange transfusion in 80%, and invasive ventilation in 80%. Forty percent received inhaled nitric oxide, and the median hospital stay was 41 days, reflecting substantial resource use.

**Conclusions:**

In this seven-year cohort, earlier presentation and pleural effusion identified antenatal NIHF pregnancies at high risk for IUFD/termination, whereas maturity at delivery was the key determinant of one-year survival in live-born NIHF. Immune hydrops showed comparatively favorable survival with fetal therapy. These data provide birth-prevalence benchmarks for a Middle East tertiary center, supporting guideline-based evaluation, timely fetal-therapy referral, and multidisciplinary care focused on targeted diagnosis and intervention, as well as safe prolongation of gestation where feasible.

## 1 Introduction

Hydrops fetalis is the pathologic accumulation of fluid in two or more fetal compartments, typically ascites, pleural effusion, pericardial effusion, or generalized skin edema (≥5 mm) (1). The etiologies of hydrops fetalis are classified as immune (red-cell alloimmunization) or non-immune (all other causes). Consistent with contemporary guidance, we defined hydrops as fluid accumulation in two or more fetal compartments. For non-immune hydrops fetalis (NIHF), we followed the Society for Maternal–Fetal Medicine (SMFM) case definition and evaluation pathway, and immune hydrops (alloimmunization) was managed according to SMFM/ACOG alloimmunization guidelines (2). The epidemiology has shifted markedly with widespread anti-D prophylaxis: immune hydrops now represents a minority, whereas NIHF accounts for approximately 80%–90% of cases, with an overall occurrence of approximately 1 in 1,700–3,800 pregnancies, depending on the population and method of diagnosis (3–5).

NIHF arises when fetal fluid homeostasis across the capillary-interstitial-lymphatic axis is disrupted, resulting in increased interstitial fluid formation and/or impaired lymphatic return (5). The etiologic spectrum is broad and heterogeneous: large reviews and guidelines consistently cite cardiovascular malformations/arrhythmias, chromosomal aneuploidy and other genetic disorders, hematologic disease (e.g., *α*-thalassemia), lymphatic dysplasia, congenital infection, and thoracic/other structural lesions among the leading causes, with a meaningful fraction remaining idiopathic despite comprehensive evaluation (2, 6, 7). The etiology and timing of presentation of hydrops fetalis are strongly associated with its prognosis, with earlier gestation at diagnosis associated with higher rates of fetal and neonatal loss (8). In carefully selected scenarios, targeted fetal therapy may modify risk. For example, in primary fetal hydrothorax, thoraco-amniotic shunting can resolve hydrops in a subset and has been associated with improved survival, although effect sizes vary by indication and the evidence base, largely retrospective series, remains heterogeneous (9–11).

Regional evidence from the Middle East is limited to small, older cohorts. For example, a single-center Saudi series from 1979 to 1994 (*n* = 17) predates contemporary diagnostic pathways and fetal therapy (12). To provide current, counseling-oriented data from our region, we undertook a seven-year, single-center cohort study at a Saudi tertiary referral unit. Using the SMFM definition of hydrops (fluid in ≥2 fetal compartments), we separated immune from non-immune cases. For NIHF, we followed the SMFM evaluation pathway, whereas immune hydrops was managed according to SMFM/ACOG alloimmunization guidance. We characterized etiologies and quantified antenatal and postnatal outcomes (termination of pregnancy [ToP], intrauterine fetal death, neonatal death, and one-year survival). For NIHF, we prespecified two analyses: (i) antenatal predictors of live birth vs. intrauterine fetal death (IUFD)/termination and (ii) among live-born NIHF, perinatal factors associated with one-year survival. We also describe management and outcomes in immune hydrops, including intrauterine transfusion.

## 2 Methods

### 2.1 Study design and setting

We performed a retrospective cohort study at King Abdul-Aziz Medical City (KAMC), Riyadh, Saudi Arabia, a tertiary maternal-fetal medicine and neonatal referral center covering the period from January 1, 2016, to December 31, 2022. The co-located fetal medicine and neonatal intensive care unit (NICU) services allowed the capture of both antenatal and postnatal data for pregnancies managed and delivered at KAMC.

### 2.2 Case identification and eligibility

Cases were ascertained by electronic searches of the fetal medicine database and neonatal medical records using diagnostic terms/codes for hydrops fetalis. Hydrops was defined antenatally as fluid in ≥2 fetal compartments (ascites, pleural effusion, pericardial effusion, or generalized skin oedema ≥5 mm), consistent with contemporary criteria. Inclusion required an antenatal diagnosis of hydrops and delivery at KAMC during the study period. Out born infants were excluded to ensure complete antenatal and neonatal information.

### 2.3 Classification and assignment of etiology

Hydrops was classified as immune or non-immune based on maternal serology and clinical context. For NIHF, etiologies were assigned using all available antenatal and postnatal information to prespecified categories: chromosomal/aneuploidy, single-gene/syndromic, cardiovascular (structural or arrhythmic), lymphatic/thoracic (e.g., congenital chylothorax), hematologic, congenital infection, urinary tract, gastrointestinal, inborn errors of metabolism, twin-to-twin transfusion syndrome, cystic hygroma/lymphatic malformation, idiopathic, and unspecified. Idiopathic NIHF was assigned when no cause was identified after a completed evaluation (detailed ultrasound and fetal echocardiography, testing for infections, and genetic studies or postnatal testing when feasible). Unspecified was used when the evaluation was incomplete (e.g., IUFD before a full work-up or when the family declined testing).

### 2.4 Antenatal evaluation and procedures

From fetal medicine records, we extracted data on the components of the work-up performed at the clinician's discretion: detailed ultrasound, fetal echocardiography, and Doppler studies, as well as invasive testing when undertaken (amniocentesis, chorionic villus sampling, or cordocentesis). Targeted procedures performed included diagnostic/therapeutic thoracocentesis. For immune hydrops, we recorded fetal blood sampling and intrauterine transfusion when performed. The gestational age (GA) at first diagnosis was recorded in weeks and subsequently grouped by trimester for descriptive analyses.

### 2.5 Perinatal and neonatal variables

For live-born infants, we collected data on the sex, GA at birth, birthweight, mode of delivery, and Apgar scores (1 and 5 min). Delivery-room and early NICU interventions were recorded, including the need for endotracheal intubation, thoracocentesis or paracentesis at birth, respiratory support modality (conventional ventilation, rescue high-frequency oscillatory ventilation [HFOV], and inhaled nitric oxide), placement of chest or abdominal drains, and exposure to blood products (packed red cells, fresh frozen plasma, and albumin). We also recorded data on culture-positive sepsis, duration of invasive ventilation, time to full enteral feeds (≥120 mL/kg/day), and length of hospital stay. For immune hydrops, data on the neonatal hematologic management, including double-volume exchange transfusion and intravenous immunoglobulin (IVIG) administration, were documented.

### 2.6 Data sources, variables, and operational definitions

Maternal, fetal, and neonatal data were extracted from the electronic medical record into a pre-piloted spreadsheet. Maternal/antenatal variables included age, parity, blood group, and antibody screening results (for immune hydrops), GA at first diagnosis of hydrops, the number and types of fluid-filled compartments, associated structural anomalies, and any invasive testing or antenatal procedures performed. Delivery/newborn variables included infant sex, GA at birth, birthweight, Apgar scores at 1 and 5 min, delivery-room interventions, early respiratory and cardiovascular support, procedures and drains, and exposure to blood products. Follow-up variables comprised survival status (*in utero*, neonatal, and up to one year), cause of death where available, duration of invasive ventilation, time to full enteral feeds, and duration of hospital stay.

Hydrops “severity” was classified based on the number of involved compartments (two, three, or four). IUFD was defined as fetal death at ≥20 weeks' gestation before delivery. Neonatal death was defined as the death of a live-born infant before hospital discharge.

### 2.7 Outcomes

For the antenatally diagnosed cohort, the primary outcomes were ToP, IUFD, neonatal death, and one-year survival. In analyses limited to live-born NIHF, the primary outcome was survival to one year. Survival status was ascertained from the electronic record, with discharge summaries and outpatient documentation reviewed when needed to confirm post-neonatal survival.

### 2.8 Ethical considerations and reporting

The King Abdullah International Medical Research Center Institutional Review Board approved the study (approval number NRC22R-532-11). The requirement for informed consent was waived due to the retrospective design and use of de-identified data. The study was reported in accordance with STROBE recommendations for observational cohort studies. Data were anonymized prior to analysis with removal of direct identifiers, and follow-up outcomes were obtained from institutional electronic medical records and verified where necessary.

### 2.9 Statistical analysis

Analyses were prespecified for two populations: (i) the antenatally diagnosed NIHF cohort, used to compare antenatal characteristics across outcome categories and to model live birth vs. IUFD/termination; and (ii) the live-born NIHF subset, used to compare perinatal characteristics by survival status and to model one-year survival.

Distributions of continuous variables were assessed using D'Agostino skewness–kurtosis statistics and inspection of histograms. Approximately normally distributed variables are summarized as the mean ± SD, skewed variables as median (IQR), and categorical variables as *n* (%). Categorical data were compared between groups using Pearson's *χ*² tests (or Fisher's exact when expected counts were less than five). For continuous data, independent-samples t-tests were used when the data were normally distributed, and the Mann–Whitney *U*-test was used otherwise. When comparing a continuous variable across more than two outcome categories, we used one-way ANOVA or a Kruskal–Wallis test as appropriate. Univariable analyses were considered exploratory, and no formal adjustment for multiple comparisons was applied; primary inferences were based on prespecified multivariable models.

For the antenatal NIHF cohort, we compared the distribution of affected compartments (ascites, skin edema, pleural effusion, and pericardial effusion), the number of involved compartments (two, three, or four), and timing of diagnosis (gestational age and trimester) across four outcomes: termination, IUFD, neonatal death, and one-year survival.

Two multivariable logistic regression models were developed. Model 1 (antenatal NIHF) used live birth vs. IUFD/termination as the dependent variable and included prespecified clinically relevant predictors, including pleural effusion, skin edema, ascites, pericardial effusion (all binary), and gestational age at diagnosis (continuous). Model 2 (live-born NIHF) used one-year survival as the dependent variable, with predictors including gestational age at delivery, Apgar scores at 1 and 5 min, gestational age at diagnosis (all continuous), and antenatal pleural effusion and skin edema (binary). Model stability was considered in relation to the number of events per variable. The results are reported as adjusted odds ratios (aORs) with 95% confidence intervals and two-sided *p*-values. As a sensitivity analysis, ridge-penalized logistic regression was performed using the same predictor sets, with results presented in the Supplementary Material.

All analyses followed a complete-case approach (denominators vary with missingness). Given the retrospective design and modest sample size, multiple imputation was not performed, and regression analyses were based on complete cases only. Statistical significance was set at *α* = 0.05. Computations were performed using IBM SPSS Statistics, version 26 (IBM Corp., Armonk, NY, USA).

## 3 Results

Over the seven years, 63,000 deliveries occurred at KAMC. We identified 116 antenatally diagnosed NIHF and 14 antenatally diagnosed immune hydrops cases, corresponding to hospital-based birth prevalences of 1.84 per 1,000 and 0.22 per 1,000 deliveries, respectively (combined 2.06 per 1,000). Among live births, 41 infants with NIHF and 10 infants with immune hydrops were delivered, yielding live born prevalences of 0.65 per 1,000 and 0.16 per 1,000 (combined 0.81 per 1,000).

Among 116 antenatally diagnosed NIHF pregnancies, the largest category was unspecified (23/116, 19.8%), followed by idiopathic (14/116, 12.1%), chromosomal abnormalities (13/116, 11.2%), congenital heart disease (12/116, 10.3%), and cystic hygroma (11/116, 9.5%) categories, and outcomes varied by etiology. Unspecified and cystic hygroma cases had no one-year survivors; most unspecified cases ended in IUFD, with the remainder electing for termination. Congenital heart disease was predominantly associated with neonatal death. In contrast, all cases of congenital chylothorax and urinary tract abnormalities, as well as both gastrointestinal cases, survived for one year. Single-gene disorders and twin-twin transfusion showed mixed outcomes, with a minority of one-year survivors. Several groups contained small numbers; therefore, the proportions should be interpreted cautiously (Table 1).

**Table 1:** Etiologies of non-immune hydrops fetalis.

Etiology	*n* (%)	IUFD	Termination	Neonatal death	Survived to 1 year
Single-gene disorder	8 (6.9)	4	0	2	2
Skeletal dysplasia	4 (3.4)	3	0	1	0
Congenital heart disease	12 (10.3)	4	0	7	1
Congenital chylothorax	3 (2.6)	0	0	0	3
Congenital thoracic malformation	3 (2.6)	2	0	1	0
Chromosomal abnormalities	13 (11.2)	5	1	6	1
Urinary tract abnormalities	3 (2.6)	0	0	0	3
Inborn errors of metabolism	7 (6.0)	4	1	1	1
Idiopathic^b^	14 (12.1)	9	0	1	3
Unspecified^a^	23 (19.8)	18	5	0	0
Infections	4 (3.4)	3	0	1	0
Twin–twin transfusion	8 (6.9)	4	0	2	2
Gastrointestinal	2 (1.7)	0	0	0	2
Vein of Galen malformation	1 (0.9)	0	0	1	0
Cystic hygroma	11 (9.5)	9	2	0	0
Total	116 (100)	65	9	23	18

a Unspecified: evaluation insufficient for etiologic assignment (most commonly IUFD before completion of work-up or limited investigations).

b Idiopathic: no cause identified despite a completed assessment according to the center's NIHF work-up. Hematologic causes, including congenital haemolytic anaemia, were included in the diagnostic framework but were not identified in this cohort.

Table 2 shows the distribution of fluid-filled compartments, antenatal procedures, and outcomes in the antenatally diagnosed NIHF cohort. Ascites was the most frequent presentation (81.0%), followed by skin edema (69.8%) and pleural effusion (64.7%); pericardial effusion was present in approximately one-third of cases (31.0%). Overall, more than half of pregnancies resulted in IUFD (56.9%), 7.8% ended in termination, and 35.3% resulted in a live birth. When outcomes were examined for the entire cohort, neonatal death attributable to NIHF occurred in 19.8% and one-year survival in 15.5%. Among the 41 live-born infants with NIHF, 43.9% were alive at one year, and 56.1% died during the neonatal period. Half of the pregnancies (50.9%) had no antenatal diagnostic or therapeutic procedure. Amniocentesis was the most common intervention (35.3%), with smaller proportions undergoing cordocentesis (11.2%), thoracocentesis (1.7%), or chorionic villus sampling (0.9%) (Table 2).

**Table 2:** Baseline characteristics of the cohort.

Domain	Variable	n/N	%
Affected compartments	Fetal ascites	94/116	81.0
	Fetal skin edema	81/116	69.8
	Fetal pleural effusion	75/116	64.7
	Fetal pericardial effusion	36/116	31.0
Overall pregnancy outcomes	Intrauterine fetal death (IUFD)	66/116	56.9
	Termination of pregnancy	9/116	7.8
	Live birth with NIHF	41/116	35.3
Outcome status recorded against full cohort	Neonatal death (NIHF)	23/116	19.8
	One-year survival (NIHF)	18/116	15.5
Outcomes among live-born NIHF only	Neonatal death (live-born NIHF)	23/41	56.1
	One-year survival (live-born NIHF)	18/41	43.9
Antenatal diagnostic or therapeutic procedures	No antenatal procedure	59/116	50.9
	Amniocentesis	41/116	35.3
	Cordocentesis	13/116	11.2
	Thoracocentesis	2/116	1.7
	Chorionic villus sampling	1/116	0.9

Data presented as median (IQR) or *n* (%).

Table 3 shows the relationship between specific fluid compartments, timing of diagnosis, and outcomes. The presence of skin edema and pleural effusion differed significantly across outcome groups (*p* = 0.036 and *p* = 0.021, respectively), whereas ascites and pericardial effusion did not (*p* = 0.507 and *p* = 0.791). Diagnosis in the third trimester was markedly more frequent among one-year survivors (88.8%), while first- and second-trimester diagnoses were more common among pregnancies ending in termination or IUFD (*p* ≤ 0.005 for each trimester comparison). Outcomes also varied by the extent of anatomic involvement: cases with two compartments had the highest proportion of one-year survivors (55.6%), whereas most of those with three or four compartments had IUFD, with very low to no one-year survival (*p* < 0.001) (Table 3).

**Table 3:** Antenatal characteristics in NIHF.

Domain	Variable	Termination	IUFD	Neonatal death	One-year survival	*p*-value
Affected compartments — % within outcome group	Fetal ascites	88.9	77.2	82.8	89.3	0.507
	Fetal skin edema	88.9	76.9	51.7	41.2	0.036
	Fetal pleural effusion	66.7	71.2	60.9	27.8	0.021
	Fetal pericardial effusion	33.3	34.8	29.1	21.4	0.791
Trimester at diagnosis — % within outcome group	First trimester	22.3	9.2	9.5	5.6	0.005
	Second trimester	44.4	55.4	28.6	5.6	0.003
	Third trimester	33.3	35.4	61.9	88.8	<0.001
Number of fluid-involved compartments — *n* (%) within row	Two compartments	1 (3.7)	7 (25.9)	4 (14.8)	15 (55.6)	<0.001
	Three compartments	5 (7.1)	45 (64.3)	17 (24.3)	3 (4.3)	<0.001
	Four compartments	3 (15.8)	14 (73.7)	2 (10.5)	0 (0.0)	<0.001

Data are presented as percentages within outcome groups; number of fluid-involved compartments is shown as *n* (row%). IUFD, intrauterine fetal death.

**Figure 1:**
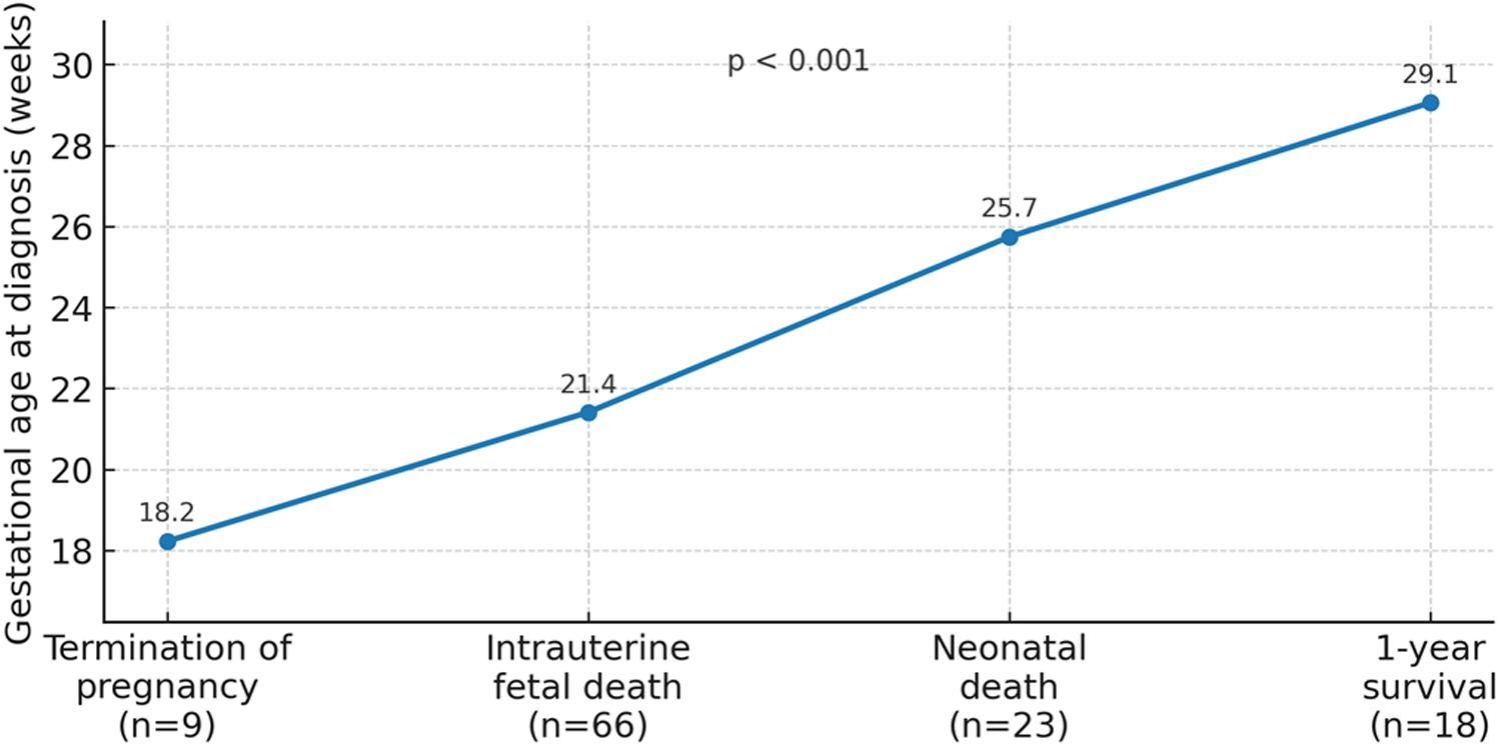
Gestational age at diagnosis by outcome (NIHF).

The mean GA at diagnosis increased progressively across outcome groups from 18.2 weeks for pregnancies ending in termination to 21.4 weeks for intrauterine fetal death, 25.7 weeks for neonatal death, and 29.1 weeks for one-year survivors. The overall difference among groups was statistically significant (*p* < 0.001) (Figure 1).

In the multivariable logistic regression modeling of IUFD/termination vs. live birth, earlier GA at diagnosis and fetal pleural effusion were independently associated with higher odds of IUFD/termination. The odds of IUFD/termination increased by approximately 20% per week of earlier diagnosis [adjusted OR (aOR) 1.20 per week of earlier diagnosis; 95% CI 1.09–1.31; *p* < 0.001]. Pleural effusion was associated with a six-fold increase in the odds of IUFD/termination (aOR 6.11; 95% CI 2.07–17.98; *p* = 0.001). Fetal skin edema (aOR 1.61; 95% CI 0.58–4.46; *p* = 0.358), ascites (aOR 2.25; 95% CI 0.58–8.76; *p* = 0.244), and pericardial effusion (aOR 2.40; 95% CI 0.81–7.14; *p* = 0.114) were not significantly associated with IUFD/termination after adjustment (Table 4).

**Table 4:** Multivariable logistic regression for IUFD/termination vs. live birth in antenatally diagnosed NIHF (*n* = 116).

Predictor	*p*-value	aOR	95% CI for aOR
Fetal pleural effusion	0.001	6.11	2.07–17.98
Gestational age at diagnosis	<0.001	1.20	1.09–1.31
Fetal skin edema	0.358	1.61	0.58–4.46
Fetal ascites	0.244	2.25	0.58–8.76
Fetal pericardial effusion	0.114	2.40	0.81–7.14
Constant	<0.001	0.001	—

Table 5 shows antenatal and birth characteristics for 41 live-born infants with NIHF stratified by one-year survival. Survivors were delivered at a later GA than non-survivors (34.4 vs. 32.8 weeks, *p* = 0.042), with a non-significant trend toward higher birthweight. GA at diagnosis did not differ significantly between groups. Antenatal pleural effusion was more frequent among non-survivors than survivors (61% vs. 28%, *p* = 0.035), whereas the frequencies of antenatal ascites and pericardial effusion did not differ significantly (Table 5).

**Table 5:** Antenatal and birth characteristics of live-born infants with NIHF by one-year survival status (*n* = 41).

Variable, Mean (SD), *n* (%)	Survivors (*n* = 18)	Non-survivors (*n* = 23)	*p*-value
Gestational age at birth (weeks)	34.4 ± 3.5	32.8 ± 4.2	0.042
Birthweight (grams)	2,523 ± 800	2,198 ± 715	0.179
Gestational age at diagnosis (weeks)	29.0 ± 6.3	26.3 ± 8.6	0.102
Male sex	10 (63)	15 (68)	0.715
Cesarean delivery	9 (53)	17 (74)	0.169
Antenatal pleural effusion	5 (28)	14 (61)	0.035
Antenatal ascites	16 (89)	19 (83)	0.572
Antenatal pericardial effusion	4 (22)	7 (30)	0.556

Table 6 shows the postnatal status at birth and the early neonatal course among live-born infants with NIHF, stratified by one-year survival. Most infants in both groups required intubation at birth, with no difference between survivors and non-survivors. Survivors had higher Apgar scores at 1 and 5 min, indicating better early postnatal condition, and a markedly longer hospital stay, consistent with survival to discharge and ongoing care needs (Table 6).

**Table 6:** Postnatal outcomes in live-born NIHF stratified by survival.

Variable *n* (%), median (IQR)	Survivors (*n* = 18)	Non-survivors (*n* = 23)	*p*-value
Need for intubation at birth	16/18 (88.9)	21/23 (91.3)	0.594
Need for thoracocentesis at birth	3/18 (16.7)	6/23 (26.0)	0.472
Need for paracentesis at birth	3/18 (16.7)	2/23 (8.7)	0.393
Apgar score at 1 min	6 (3)	2 (2)	< 0.001
Apgar score at 5 min	7 (2)	4 (3)	0.001
Rescue HFOV	3/18 (16.7)	7/23 (30.4)	0.369
Inhaled nitric oxide	5/18 (27.8)	9/23 (39.1)	0.447
Chest tube placement	4/18 (22.2)	8/23 (34.8)	0.380
Need for abdominal drain	4/18 (22.2)	3/23 (13.0)	0.438
Culture-positive sepsis	1/18 (5.6)	2/23 (8.7)	0.702
Duration of invasive ventilation (days)	11 (4, 24)	10 (3, 30)	0.359
Length of hospital stay (days)	44 (26, 72)	24 (16, 52)	<0.001

Table 7 shows the multivariable logistic regression for one-year survival among the 41 live-born infants with NIHF. The model included GA at delivery and at diagnosis, Apgar scores at 1 and 5 min, and antenatal pleural effusion and skin edema. After adjustment, GA at delivery remained the only independent predictor of one-year survival, with a 55% increase in the odds of survival for each additional week of gestation at birth (aOR 1.55; 95% CI 1.09–2.22; *p* = 0.016) (Table 7).

**Table 7:** Multivariable analysis of predictors of survival.

Predictor	*p*-value	aOR	95% CI for aOR
Fetal skin edema	0.951	1.06	0.17–6.83
Fetal pleural effusion	0.407	2.36	0.31–17.99
Gestational age at delivery	0.016	1.55	1.09–2.22
Apgar score at 1 min	0.715	1.18	0.48–2.92
Apgar score at 5 min	0.408	1.45	0.60–3.51
Gestational age at diagnosis	0.651	0.96	0.82–1.13
Constant	0.005	0.000	—

Table 8 shows the maternal, antenatal, and perinatal profile of the immune hydrops cohort (*n* = 14; postnatal data available for 10 live-born infants). Maternal alloimmunization mostly involved multiple antibodies (6/14, 42.9%), followed by anti-D alone (4/14, 28.6%); anti-Kell, anti-C, and anti-E were less frequent (2/14, 1/14, and 1/14, respectively). Hydrops was typically diagnosed in the late second to early third trimester (median 27.3 weeks). Invasive fetal therapy was often performed. Among cases with documented invasive management, cordocentesis with intrauterine transfusion was performed in 9/10 (90%). Live-born infants were delivered preterm (median 32.4 weeks), with a median birthweight of approximately 2.3 kg. The immediate postnatal condition reflected moderate compromise (the median Apgar score was 4 at 1 min and 7 at 5 min), and delivery-room drainage was required in a minority (thoracocentesis 2/10 [20%] and paracentesis 1/10 [10%]). Overall, the one-year survival rate was 71.4% (10/14) in this immune hydrops cohort (Table 8).

**Table 8:** Characteristics and outcomes in immune hydrops fetalis.

Domain	Variable	Median (IQR), *n* (%)
Maternal	Maternal age years	31.5 (24, 40)
Maternal alloantibody specificity	Anti-D	4/14 (28.6%)
	Anti-Kell	2/14 (14.3%)
	Anti-C	1/14 (7.1%)
	Anti-E	1/14 (7.1%)
	Multiple antibodies (combinations)	6/14 (42.9%)
Antenatal	Gestational age at diagnosis, weeks	27.3 (18, 33)
	Cordocentesis with intrauterine transfusion (IUT)^a^	9/10 (90%)
Delivery/newborn	Gestational age at delivery, weeks	32.4 (29, 35)
	Birthweight, g	2,284 (1,650, 3,160)
	Male sex	5/10 (50%)
	Caesarean delivery	5/10 (50%)
	Apgar score at 1 min	4 (1, 8)
	Apgar score at 5 min	7 (4, 9)
Delivery-room procedures	Thoracocentesis at birth	2/10 (20%)
	Paracentesis at birth	1/10 (10%)
Outcome	One-year survival	10/14 (71.4%)

Denominators differ where only live-born infants had postnatal data (*n* = 10).

a Denominator reflects cases with an antenatal invasive therapy record; the 9/10 indicates IUT among those who underwent cordocentesis.

Table 9 shows the postnatal course of the ten live-born infants with immune hydrops. Disease-directed hematologic therapy was universally or near-universally employed, with IVIG administration in 10/10 (100%) and double-volume exchange transfusion in 8/10 (80%). Respiratory support requirements were substantial: 8/10 (80%) required invasive mechanical ventilation, and 4/10 (40%) were administered inhaled nitric oxide; the median duration of ventilation was 13.5 days. Drainage procedures were frequent, with chest tubes inserted in 5/10 (50%) and abdominal drains inserted in 3/10 (30%) infants. Nutritional progression was prolonged, requiring a median of 24 days to reach full enteral feeding. Ongoing hematologic support was common, with medians of four packed red cells, five fresh frozen plasma, and three albumin transfusions per infant during admission. The overall resource use is reflected in a median hospital stay of 41 days (Table 9).

**Table 9:** Postnatal course of live-born infants with immune hydrops fetalis (*n* = 10).

Outcome/Intervention	n (%), Median (IQR)
Double-volume exchange transfusion	8/10 (80)
Intravenous immunoglobulin (IVIG) administration	10/10 (100)
Invasive mechanical ventilation	8/10 (80)
Inhaled nitric oxide (iNO) administration	4/10 (40)
Duration of invasive ventilation (days)	13.5 (2, 40)
Chest tube placement	5/10 (50)
Abdominal drain placement	3/10 (30)
Time to full enteral feeding (days)	24 (14, 53)
Packed red cell transfusions (per infant)	4 (1, 10)
Fresh frozen plasma transfusions (per infant)	5 (1, 16)
Albumin transfusions (per infant)	3 (1, 12)
Length of hospital stay (days)	41 (25, 82)

## 4 Discussion

In this single center retrospective study, we characterized the etiologies, antenatal and postnatal courses, and one year survival rates of fetuses with hydrops fetalis managed at a tertiary referral center and identified antenatal predictors of these outcomes. This cohort, one of the larger single center series reported from the Middle East, separates immune from non-immune hydrops and evaluates its multivariable predictors tailored to two decision points. Over the seven years encompassing 63,000 deliveries at our institution, the hospital based prevalences of hydrops fetalis were 1.84 per 1,000 for antenatally diagnosed NIHF and 0.22 per 1,000 for immune hydrops; among live births, the prevalences were 0.65 per 1,000 (NIHF) and 0.16 per 1,000 (immune hydrops). These figures reflect the case mix seen at a tertiary referral center and should not be interpreted as the prevalence within the general population. In the antenatal NIHF cohort, earlier GA at diagnosis and pleural effusion independently increased the odds of IUFD or termination. In contrast, among live born NIHF infants, GA at delivery was the sole independent predictor of one year survival. Immune hydrops, predominantly managed with intrauterine transfusion, had a relatively favorable survival rate. These findings, particularly those related to gestational age, should be interpreted within the context of underlying disease severity and clinical decision making. Gestational age at diagnosis and delivery may be influenced by these factors, introducing potential confounding by indication. In addition, intrauterine fetal death and termination of pregnancy represent competing antenatal outcomes that may have distinct underlying mechanisms. While modeling these outcomes separately using multinomial or competing risk approaches may provide additional insight, such analyses were not performed due to the modest sample size and exploratory nature of this study.

These findings should be interpreted within the context of contemporary guidance. We adhered to the SMFM definition of NIHF (≥ two abnormal fetal fluid collections) (2) and used a structured etiological work-up spanning chromosomal, genetic, cardiac, hematologic, infectious, lymphatic/thoracic, and other structural causes. As in guideline summaries and large reviews, NIHF predominated over immune hydrops, and the aetiologies were heterogeneous (2, 5, 13). Outcomes in NIHF are strongly influenced by the underlying etiology. However, the relatively small numbers within individual etiologic subgroups limited the ability to perform robust stratified or grouped analyses without introducing unstable estimates. Although formal subgroup analysis was not feasible, the direction of the main associations observed, including earlier gestational age at diagnosis and the presence of pleural effusion, is likely to be relevant across multiple etiologic categories. Further studies with larger cohorts are needed to evaluate potential effect modification by etiology. Our data offer a pragmatic framework for counselling in tertiary centers: (i) the timing of presentation is prognostically important, with earlier GA at diagnosis independently signaling a higher risk of IUFD/termination; (ii) thoracic involvement, particularly pleural effusion, identifies a subgroup at elevated antenatal risk and potential candidacy for targeted fetal therapy when appropriate; and (iii) among live-born infants with NIHF, GA at delivery is the primary determinant of one-year survival, supporting the implementation of care plans that safely prolong gestation where feasible (14).

Among pregnancies with NIHF in our series, the combined rate of IUFD and ToP was 74/116 (63.8%), indicating substantial antenatal non-viability. Our ToP proportion alone (7.8%) is lower than that of several contemporary cohorts that report ToP rates of approximately 44%–51% and 73% when a prenatal genetic diagnosis is established, with earlier GA at diagnosis also linked to a higher likelihood of ToP (8, 15–18). These differences likely reflect variations in legal access to termination, local clinical practices and policies, patient and family preferences, referral patterns, availability of fetal therapy, and timing of diagnosis. In settings where access to termination is limited or decisions occur later, a greater proportion of non-viable pregnancies may result in IUFD rather than ToP. Consistent with these reports, our multivariable analysis showed that earlier GA at diagnosis was strongly associated with the composite endpoint of IUFD/ToP, supporting the interpretation that both timing of presentation and underlying etiology are key determinants of antenatal outcome, whereas the relative distribution of IUFD and ToP is influenced by health system and contextual factors.

Among live-born infants with NIHF in our cohort, the one-year mortality rate was 56%, consistent with high mortality reported elsewhere (19, 20). This risk likely reflects a combination of prematurity at delivery, the severity of underlying aetiologies, and the physiological burden of multi-compartment fluid accumulation that limits adequate ventilation. These findings also highlight the substantial resource utilization in this population, including high rates of respiratory support, drainage procedures, and prolonged hospitalization. In our study, survivors were delivered at a later gestational age, and gestational age at delivery remained the only independent predictor of one-year survival. Taken together, these findings emphasize the central role of maturity at delivery in shaping outcomes and support management strategies aimed at safely prolonging pregnancy where feasible.

The strong relationship we observed between earlier GA at diagnosis and adverse outcome fits with that reported in previous studies. In a large UK series that stratified participants by timing of diagnosis, earlier diagnosis was associated with higher perinatal loss, and in cases managed expectantly without fetal intervention, GA at diagnosis was the only factor associated with mortality (15). Similarly, other studies have reported worse outcomes with earlier presentations and a more extensive disease burden (8, 21, 22). Biologically, early-onset hydrops often reflects lethal chromosomal or structural anomalies, severe genetic syndromes, or intractable pathophysiology. In contrast, later-onset presentations may include potentially reversible entities, allowing the fetus to mature.

Pleural effusion independently predicted IUFD/termination in our antenatal model. This likely indicates advanced thoracic compromise or underlying lymphatic dysplasia. The literature suggests that the prognosis in primary hydrothorax depends on the disease's pathophysiology and the feasibility of fetal therapy. Selected fetuses improve following pleuro-amniotic shunting, with survival approaching 60% in some series, although outcomes vary across studies (9). Our finding that primary chylothorax among live-born infants had favorable survival is compatible with reports of good outcomes when thoracic drainage and nutritional strategies are effective (with or without prenatal shunting) (9).

A sizeable fraction of NIHF cases in our cohort remained “unspecified.” This largely reflects IUFD occurring before a complete diagnostic work up could be undertaken, limiting cytogenetic, molecular, and postmortem evaluation. Additional contributors included resource and turnaround time constraints early in the study period, as well as occasional parental refusal of invasive testing or autopsy. Despite extensive research, the aetiology of NIHF remains unknown in approximately 25% of cases (23, 24). During the study period, genetic evaluation evolved with increasing use of chromosomal microarray and, more recently, exome sequencing; however, uptake and turnaround times were variable and not consistently captured in this retrospective dataset. Expanding genetic evaluation through routine chromosomal microarray combined with exome sequencing, and, where feasible, targeted testing of fetal or placental tissue following IUFD, together with a structured post-IUFD evaluation pathway incorporating systematic placental pathology, may reduce the proportion of unspecified cases, refine recurrence risk counselling, and reclassify cases currently labeled as unspecified.

Our etiologic distribution mirrors prior reviews highlighting the predominance of cardiovascular and chromosomal causes among classified NIHF and the persistent proportion of idiopathic or unspecified cases, particularly when IUFD limits evaluation (13, 14). Regional data from the Middle East show a similar case-mix and variability in outcomes, influenced by referral patterns, access to fetal therapy, and diagnostic practices. A historical Saudi cohort (1979–1994; *n* = 17) reported high mortality and limited use of fetal therapy (12), whereas a more recent Qatari study (2003–2011; *n* = 64) identified cardiac and chromosomal etiologies as the most frequent and reported a live-born survival of approximately 59% (25). These findings are broadly comparable to our one-year survival rates for antenatally diagnosed NIHF (15.5%) and live-born NIHF (43.9%), allowing for differences in follow-up duration and case mix.

Because the availability of interventions can shape outcomes of hydrops, we clarify the fetal therapy provided in our center. In the immune subgroup, cordocentesis with intrauterine transfusion was routinely available and performed in most invasively managed pregnancies, which likely contributed to the observed survival. In NIHF, diagnostic/therapeutic thoracocentesis was available but applied selectively. Pleuro-amniotic shunting was considered on a case-by-case basis rather than as a standard therapy. This context helps interpret the balance of outcomes across etiologies and explains differences from centers where thoracic interventions are more commonly performed.

The immune hydrops subgroup in our study achieved a one-year survival rate of approximately 71% with intrauterine transfusion-based management, consistent with contemporary experience in alloimmune anemia, in which IUT has transformed outcomes compared with the pre-prophylaxis era (2). These data support the need for rapid referral to centers with fetal therapy capability. Similarly, a single-center Chinese cohort study of severe hemolytic disease of the fetus and newborn (HDFN) treated with IUT (81 fetuses) reported high overall survival rates (fetal 90%, neonatal 98.6%) but markedly lower survival in hydropic fetuses (61%), with a frequent need for exchange and late top-up transfusions supporting timely fetal therapy and structured postnatal follow-up (26).

This study has several strengths and certain limitations. The strengths include the comprehensive capture of antenatal, delivery room, and neonatal data within a single care pathway, the separation of immune and non-immune hydrops, and prespecified multivariable analyses focused on clinically actionable predictors. The limitations include the retrospective design, modest numbers within several etiologic strata, the inherent selection and referral bias of a tertiary center, incomplete etiologic work up before IUFD, and lack of data on neurodevelopmental outcomes of survivors. Given the presence of missing data, complete case analysis may introduce bias if data were not completely at random; however, this approach was considered appropriate given the study design and sample size. The multivariable models were exploratory and not intended for clinical prediction tool development. Given the modest sample size and limited model stability, particularly in the live born NIHF cohort, the events per variable ratio was low and estimates were attenuated after penalization, indicating potential overfitting. More advanced internal validation methods, including bootstrap validation, were not performed; however, penalized regression (ridge) was explored as a sensitivity analysis and is presented in the Supplementary Material. Accordingly, derivation of a bedside risk tool or decision curve analysis was not appropriate, and external validation in larger cohorts is required before clinical implementation. We deliberately chose one year survival as the primary endpoint because most mortality in hydrops occurs *in utero*, during the neonatal period, or in early infancy. Longer term outcomes remain important but require prospective follow up beyond the scope of this study.

## 5 Conclusion

In this single-center cohort from a tertiary referral hospital, NIHF accounted for most antenatally diagnosed cases and was associated with substantial fetal and neonatal mortality. Two directly actionable points for counseling emerged: an earlier GA at diagnosis and the presence of pleural effusion independently increased the odds of intrauterine loss or termination, whereas among live-born infants with NIHF, gestational maturity at delivery was the sole independent determinant of one-year survival. Immune hydrops showed comparatively favorable outcomes with timely intrauterine transfusion. Taken together, these findings support a structured evaluation pathway, early referral to centers with fetal therapy capability, and multidisciplinary management aimed at (i) identifying etiologies with targeted testing, (ii) considering fetal intervention in selected thoracic pathologies, and (iii) safely prolonging gestation when feasible. Reducing the proportion of “unspecified” NIHF through the broader use of contemporary genetic diagnostics should refine recurrence counseling and prognostication in our setting and similar tertiary networks.

## Data Availability

The raw data supporting the conclusions of this article will be made available by the authors, without undue reservation.

## References

[1] Skoll MA Sharland GK Allan LD. Is the ultrasound definition of fluid collections in non-immune hydrops fetalis helpful in defining the underlying cause or predicting outcome? Ultrasound Obstet Gynecol. (1991) 1:309–12. 10.1046/j.1469-0705.1991.01050309.x 12797034

[2] Norton ME Chauhan SP Dashe JS. Society for maternal-fetal medicine (SMFM) clinical guideline #7: nonimmune hydrops fetalis. Am J Obstet Gynecol. (2015) 212:127–39. 10.1016/j.ajog.2014.12.018 25557883

[3] Al-Kouatly HB Shivashankar K Mossayebi MH Makhamreh M Critchlow E Gao Z Diagnostic yield from prenatal exome sequencing for non-immune hydrops fetalis: a systematic review and meta-analysis. Clin Genet. (2023) 103:503–12. 10.1111/cge.14309 36757664

[4] Makhamreh MM Shivashankar K Araji S Critchlow E O'Brien BM Wodoslawsky S RASopathies are the most common set of monogenic syndromes identified by exome sequencing for nonimmune hydrops fetalis: a systematic review and meta-analysis. Am J Med Genet A. (2024) 194:63494. 10.1002/ajmg.a.63494 38156365

[5] Bellini C Hennekam RC. Non-immune hydrops fetalis: a short review of etiology and pathophysiology. Am J Med Genet A. (2012) 158A:597–605. 10.1002/ajmg.a.34438 22302731

[6] Sparks TN Thao K Lianoglou BR Boe NM Bruce KG Datkhaeva I Nonimmune hydrops fetalis: identifying the underlying genetic etiology. Genet Med. (2019) 21:1339–44. 10.1038/s41436-018-0352-6 30410095 PMC6509016

[7] Mardy AH Chetty SP Norton ME Sparks TN. A system-based approach to the genetic etiologies of non-immune hydrops fetalis. Prenat Diagn. (2019) 39:732–50. 10.1002/pd.5479 31087399 PMC6699893

[8] Reischer T Muth B Catic A Monod C Linder T Göbl C Clinical course and outcome of non-immune fetal hydrops in singleton pregnancies. J Clin Med. (2022) 11:702. 10.3390/jcm11030702 35160154 PMC8836777

[9] Yinon Y Grisaru-Granovsky S Chaddha V Windrim R Seaward PGR Kelly EN Perinatal outcome following fetal chest shunt insertion for pleural effusion. Ultrasound Obstet Gynecol. (2010) 36:58–64. 10.1002/uog.7507 20069656

[10] Petersen S Kaur R Thomas JT Cincotta R Gardener G. The outcome of isolated primary fetal hydrothorax: a 10-year review from a tertiary center. Fetal Diagn Ther. (2013) 34:69–76. 10.1159/000351855 23817182

[11] Carson E Devaseelan P Ong S. Systematic review of pleural-amniotic shunt insertion vs. Conservative management in isolated bilateral fetal hydrothorax without hydrops. Ir J Med Sci. (2020) 189:595–601. 10.1007/s11845-019-02094-5 31745722

[12] Rejjal ARA Rahbeeni Z Al-Zahrani A-F. Prognostic factors and prenatal management in non immune hydrops fetalis are still a dilemma. J Perinat Med. (1996) 24:461–66. 10.1515/jpme.1996.24.5.461 8950726

[13] Bellini C Donarini G Paladini D Calevo MG Bellini T Ramenghi LA Etiology of non-immune hydrops fetalis: an update. Am J Med Genet A. (2015) 167:1082–8. 10.1002/ajmg.a.36988 25712632

[14] Meng D Li Q Hu X Wang L Tan S Su J Etiology and outcome of non-immune hydrops fetalis in southern China: report of 1004 cases. Sci Rep. (2019) 9:10726. 10.1038/s41598-019-47050-6 31341179 PMC6656761

[15] Sileo FG Kulkarni A Branescu I Homfray T Dempsey E Mansour S Non-immune fetal hydrops: etiology and outcome according to gestational age at diagnosis. Ultrasound Obstet Gynecol. (2020) 56:416–21. 10.1002/uog.22019 32196790

[16] Choi H Van Riper M Thoyre S. Decision making following a prenatal diagnosis of down syndrome: an integrative review. J Midwifery Womens Health. (2012) 57:156–64. 10.1111/j.1542-2011.2011.00109.x 22432488

[17] Natoli JL Ackerman DL McDermott S Edwards JG. Prenatal diagnosis of down syndrome: a systematic review of termination rates (1995–2011). Prenat Diagn. (2012) 32:142–53. 10.1002/pd.2910 22418958

[18] Ozyuncu O Orgul G Tanacan A Aktoz F Guleray N Fadiloglu E Retrospective analysis of indications for termination of pregnancy. J Obstet Gynaecol. (2019) 39:355–8. 10.1080/01443615.2018.1506427 30428730

[19] Swearingen C Colvin ZA Leuthner SR. Nonimmune hydrops fetalis. Clin Perinatol. (2020) 47:105–21. 10.1016/j.clp.2019.10.001 32000919

[20] Nassr AA Ness A Hosseinzadeh P Salmanian B Espinoza J Berger V Outcome and treatment of antenatally diagnosed nonimmune hydrops fetalis. Fetal Diagn Ther. (2018) 43:123–8. 10.1159/000475990 28647738

[21] Fukushima K Morokuma S Fujita Y Tsukimori K Satoh S Ochiai M Short-term and long-term outcomes of 214 cases of non-immune hydrops fetalis. Early Hum Dev. (2011) 87:571–5. 10.1016/j.earlhumdev.2011.04.015 21592689

[22] Steurer MA Peyvandi S Baer RJ MacKenzie T Li BC Norton ME Epidemiology of live born infants with nonimmune hydrops fetalis—insights from a population-based dataset. J Pediatr. (2017) 187:182–8.e3. 10.1016/j.jpeds.2017.04.025 28533037

[23] Bellini C Hennekam RCM Fulcheri E Rutigliani M Morcaldi G Boccardo F Etiology of nonimmune hydrops fetalis: a systematic review. American J of Med Genetics Pt A. (2009) 149A:844–51. 10.1002/ajmg.a.32655 19334091

[24] Santo S Mansour S Thilaganathan B Homfray T Papageorghiou A Calvert S Prenatal diagnosis of non-immune hydrops fetalis: what do we tell the parents? Prenat Diagn. (2011) 31:186–95. 10.1002/pd.2677 21268039

[25] Hasnani-Samnani Z Mahmoud MIM Farid I Al Naggar E Ahmed B. Non-immune hydrops: qatar experience. J Matern Fetal Neonatal Med. (2013) 26:449–43. 10.3109/14767058.2012.733781 23039279

[26] Pan W Wu H Chen J Mo X Wang H Fang Q Fetal and neonatal outcome in severe alloimmunization managed with intrauterine transfusion: 18-year experience in a tertiary referral hospital in China. Front Pediatr. (2023) 11:1157004. 10.3389/fped.2023.1157004 37124190 PMC10130633

