# Supplementary material for: Immune and non-immune hydrops fetalis in a Saudi tertiary center: etiologies, antenatal predictors, perinatal outcomes, and one-year survival in a seven-year cohort
Source: Front Pediatr. 2026 May 4;14:1693325. doi: 10.3389/fped.2026.1693325 (PMC13180879; doi:10.3389/fped.2026.1693325)
Supplement: Supplementary file 1 [file Supplementaryfile1.docx]

**Supplementary File**

**Supplementary analysis: Ridge-penalised logistic regression**

This supplementary analysis was conducted to assess potential overfitting in the multivariable logistic regression models by applying ridge-penalized regression.

**Model 1: Antenatal NIHF outcome (IUFD/termination vs live birth)**

In the antenatal NIHF model, ridge-penalized regression demonstrated preservation of the direction of key associations. Pleural effusion remained associated with increased odds of IUFD or termination, while higher gestational age at diagnosis remained associated with lower risk. As expected, effect sizes were attenuated toward the null, consistent with coefficient shrinkage.

**Model 2: Live-born NIHF survival (one-year survival)**

In the live-born NIHF survival model, ridge-penalized regression resulted in attenuation of effect estimates. Several predictors, including pleural effusion and skin oedema, showed marked reduction toward null effects. This finding is consistent with limited model stability and supports cautious interpretation of the primary regression results.

Overall, the ridge-penalized sensitivity analysis suggests that the antenatal NIHF model findings are directionally consistent after penalization, whereas the survival model shows greater instability, likely reflecting modest event counts. These findings should be interpreted in the context of the exploratory nature of the models.
