# Supplementary figures and images for: Immune and non-immune hydrops fetalis in a Saudi tertiary center: etiologies, antenatal predictors, perinatal outcomes, and one-year survival in a seven-year cohort
Source: Front Pediatr. 2026 May 4;14:1693325. doi: 10.3389/fped.2026.1693325 (PMC13180879; doi:10.3389/fped.2026.1693325)

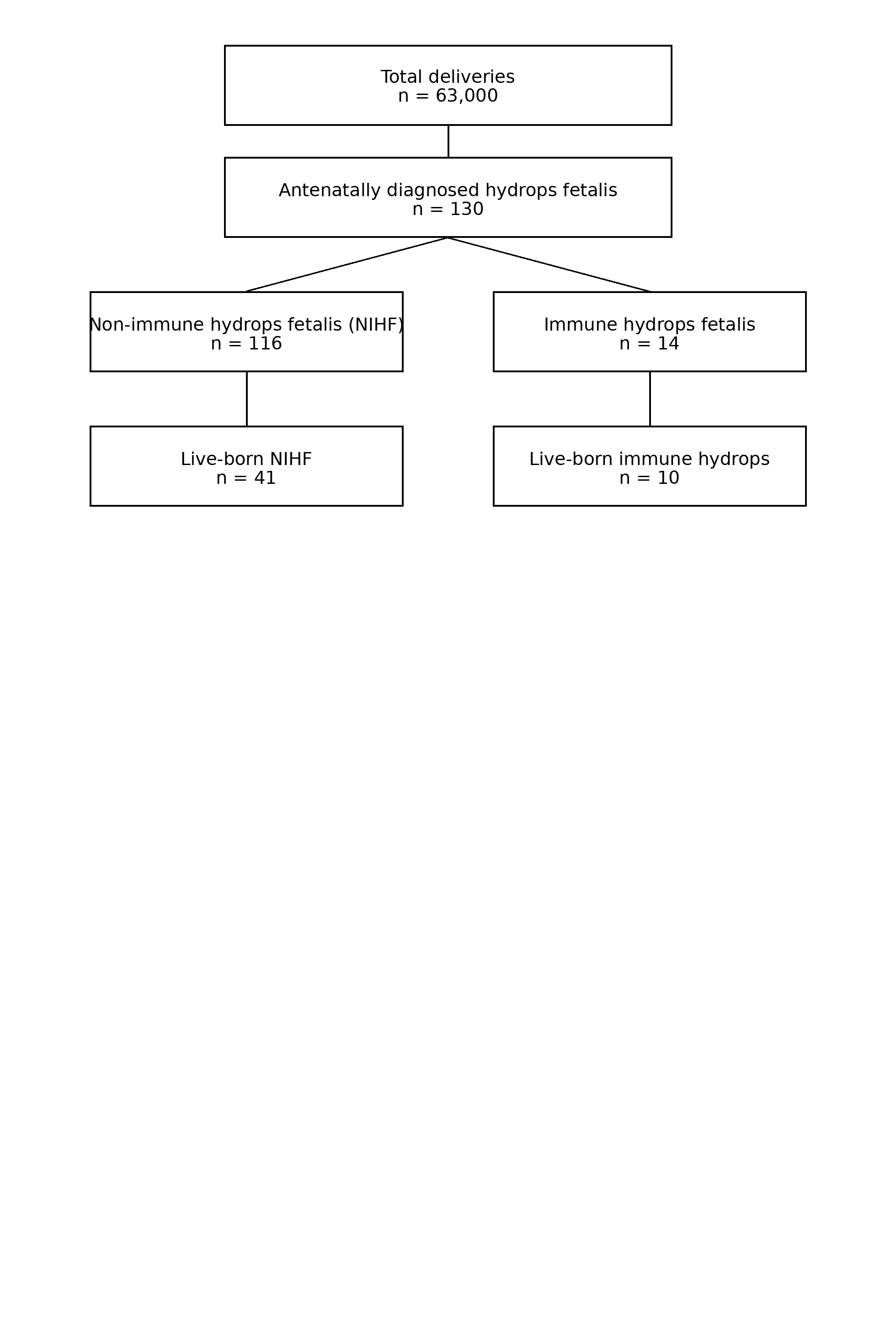

Supplement: Supplementary file 2 [file Image1.tiff]
